# The Clinical Features, Risk Factors, and Outcome of Aneurysmal Lesions in Behcet's Disease

**DOI:** 10.1155/2019/9198506

**Published:** 2019-12-20

**Authors:** Jiaxin Zhou, Jing Shi, Jinjing Liu, Luxi Sun, Lu Li, Chaoran Li, Xiuhua Wu, Yining Wang, Xinping Tian, Xiaofeng Zeng, Yi Liu, Wenjie Zheng, Yan Zhao

**Affiliations:** ^1^Department of Rheumatology, Peking Union Medical College Hospital, Chinese Academy of Medical Sciences, Peking Union Medical College, National Clinical Research Center for Dermatologic and Immunologic Deseases (NCRC-DID), The Ministry of Education Key Laboratory, Beijing, China; ^2^Department of Radiology, Peking Union Medical College Hospital, Beijing 100730, China; ^3^Department of Rheumatology, West China Hospital, Sichuan University, Guoxue Xiang 37, Chengdu, Sichuan 610041, China

## Abstract

**Objective:**

To investigate the clinical features and potential risk factors of aneurysmal lesions in Behcet's disease (BD).

**Methods:**

We retrospectively reviewed the clinical data of BD patients with aneurysmal lesions in our institute from 1997 to 2017 and compared them with 207 BD patients without aneurysmal lesions. The treatment and outcome of these patients were also analyzed.

**Results:**

Sixty-nine patients were included with 117 aneurysmal lesions. The average period between BD onset and diagnosis of aneurysmal lesion was 5.4 ± 5.5 years. Thirty-three patients (47.8%) had multiple aneurysmal lesions. Ten patients developed 20 pulmonary artery aneurysms alone. For the other 97 aortic and/or peripheral artery aneurysms in 59 patients, the most commonly affected vessels were abdominal aorta (27/97, 27.8%), coronary artery (10/97, 10.3%), and superficial femoral artery (8/97, 8.2%). Multivariate analysis revealed pathergy reaction (OR = 3.78 (1.70-8.41)), arterial stenosis or occlusion (OR = 44.12 (11.56-168.35)), and arterial thrombosis (OR = 9.27 (2.33-36.93)) as independent predictors of aneurysmal lesions in BD. With a mean follow-up of 5.5 ± 4.0 years, 40 patients (58.0%) achieved clinical improvements, 15 patients (21.7%) relapsed, and 10 patients (14.5%) died. The respective estimated cumulative 1- and 5-year relapse-free rates were 91.3% and 76.3%, and the respective estimated 1- and 5-year survival rates were 95.0% and 87.2%.

**Conclusion:**

Aneurysmal lesions are severe complications in BD. Pathergy reaction, arterial stenosis or occlusion, and arterial thrombosis are the risk factors of aneurysmal lesions in BD. Achieving BD remission and performing surgical or interventional procedures are both important in the treatment of these patients.

## 1. Introduction

Behcet's disease (BD) is a systemic disease characterized by recurrent oral and/or genital aphthous ulcers and skin lesions, accompanied by multiple organ involvement. According to the 2012 Revised International Chapel Hill Consensus Conference Nomenclature of Vasculitides, BD is classified as variable vessel vasculitis that can affect vessels of any size (small, medium, and large) and type (arteries, veins, and capillaries) [1]. Arterial lesions, including arterial occlusions, stenosis, and aneurysmal lesions, are relatively rare complications in BD patients [2–4], but correlate closely with patient's mortality and morbidity due to postoperative complications and life-threatening risks. To systemically analyze the clinical characteristics, risk factors, and prognosis of BD patients complicated with aneurysmal lesions, we conducted a retrospective study of BD patients who were admitted to a single center in China, including 69 patients identified with aneurysmal lesions. We summarized the clinical characteristics, treatment, and outcome and further explored the potential risk factors for aneurysmal lesions in BD patients.

## 2. Patients and Methods

### 2.1. Patients

BD patients who were admitted to Peking Union Medical College Hospital between October 1997 and April 2017 were retrospectively enrolled. All patients fulfilled the 1990 International Study Group (ISG) criteria [5] or the 2013 International Criteria for Behcet's Disease (ICBD) criteria [6]. The diagnosis of aneurysmal lesions was based on clinical manifestations and imaging findings, including Doppler ultrasonography, computed tomography with or without angiography, magnetic resonance imaging with angiography, and digital subtraction angiography. An aneurysmal lesion is defined as a permanent, localized dilation of an artery having at least 50% increase in diameter compared to the expected normal size of the artery. Posttraumatic or postprocedural aneurysms were routinely excluded. The aneurysmal lesions were divided into aortic and/or peripheral aneurysmal lesions and pulmonary artery aneurysms (PAA). The aortic and/or peripheral aneurysmal lesions were further classified into true aneurysm and pseudoaneurysm (false aneurysm). True aneurysm is diagnosed when the wall of the aneurysm consists of the original arterial wall. Pseudoaneurysm is the aneurysmal lesion that has no artery wall structure [7]. The site, type, and number of aneurysmal lesions were thoroughly recorded and analyzed. Patients' other clinical characteristics, demographics, laboratory results, treatment, and outcome were all reviewed. Other vascular involvements, including arterial stenosis or occlusion, venous thrombosis, arterial thrombosis, and venous stenosis or occlusion, were also studied. For each patient with aneurysmal lesions, 3 age- and gender-matched BD patients without aneurysmal lesions were randomly selected and served as controls.

This study complied with the Declaration of Helsinki and was reviewed and approved by the institutional ethics review board of Peking Union Medical College Hospital (S-443). Given the study was based on the review of medical records, written informed consent was waived.

### 2.2. Statistical Analysis

Categorical variables were represented as frequencies and percentages and were compared with Chi-square analysis. Normal and nonnormally distributed quantitative variables were expressed as mean ± SD and median (range) and were compared using the unpaired *t*-test and Mann–Whitney *U* test, respectively. Univariate logistic regression analysis was used to evaluate candidate predictors of developing aneurysmal lesions, and variables with the *P* value < 0.01 were further included in the multivariate analysis. A conditional forward stepwise multivariate stepwise analysis was used to confirm the risk factors for aneurysmal formation in BD patients. A two-sided *P* value < 0.05 was defined as a statistically significant difference. Survival and cumulative risk of relapse analysis were estimated using the Kaplan-Meier method. All data were assessed on the computer using an SPSS 22.0 software package.

## 3. Results

### 3.1. Demographics

A total of 69 hospitalized BD patients with aneurysmal lesions were enrolled, suggesting a male predominance (56 males, 13 females). Among them, all patients fulfilled the 2013 ICBD criteria and 50 patients (72.5%) fulfilled the 1990 ISG criteria. The mean age at the onset of BD was 29.9 ± 11.8 years, and the average interval between BD onset and detection of the aneurysmal lesion was 5.4 ± 5.5 (range 0-27.7) years. Aneurysmal lesions as the initial presentation were observed in 3 BD patients.

### 3.2. Characteristics of Aneurysmal Lesions

A total of 117 aneurysmal lesions were detected in the 69 patients, including 97 (82.9%) aortic and/or peripheral artery aneurysmal lesions and 20 (17.1%) PAA. Thirty-three patients (47.8%) had multiple aneurysmal lesions, and 12 patients (17.4%) had 3 or more lesions.

**Figure 1:**
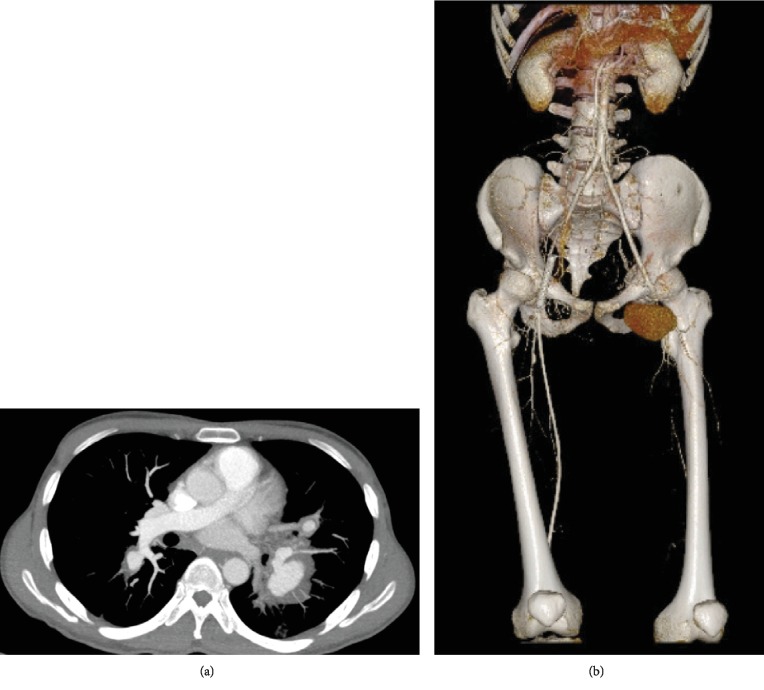
(a) Contrast-enhanced computed tomography of pulmonary artery showed a 33-year-old male BD patient with multiple pseudoaneurysms in bilateral pulmonary arteries. (b) Computed tomography with angiography of lower extremities showed a 19-year-old male BD patient with pseudoaneurysm on the left superficial femoral artery.

Notably, 10 patients (14.5%) developed pulmonary artery aneurysms alone, and all manifested with hemoptysis (Figure 1(a)). For the other 97 aortic and/or peripheral artery aneurysms in 59 patients, they can be further classified into 26 (26.8%) true aneurysms and 67 (69.1%) pseudoaneurysms and the last 4 (4.1%) aneurysms were unable to classify due to lack of enough data on the report. The most commonly affected aortic and/or peripheral vessel was abdominal aorta (27/97, 27.8%), followed by coronary artery (10/97, 10.3%), superficial femoral artery (8/97, 8.2%) (Figure 1(b)), subclavian artery (5/97, 5.2%), and popliteal artery (5/97, 5.2%). The site, number, and type of each aortic and/or peripheral aneurysmal lesion are summarized in Table 1.

For the peripheral aneurysmal lesions, pain, swelling, and palpable masses of the affected site were the most common manifestations. Lower gastrointestinal bleeding was presented in the patient with ileocolic artery pseudoaneurysm. For patients with aortic aneurysms, abdominal pain and chest pain were the most common manifestations. Two patients were asymptomatic and diagnosed incidentally.

Coronary artery involvement was observed in 7 patients (10.1%) with a mean age of 30.5 ± 11.3 years. Six of them were diagnosed by coronary angiography, and the last patient was diagnosed by CT angiography of the coronary artery. Among them, 4 patients were presented as unstable angina pectoris, 2 patients had an acute myocardial infarction, and progressed dyspnea was presented in 1 patient.

Additionally, 48 patients (69.6%) were also detected with one or more other types of vascular involvement. The most prominent involvement was arterial stenosis or occlusion (26 cases, 37.7%), including subclavian artery (9 cases), external iliac artery (7 cases), celiac truck (6 cases), coronary artery (5 cases), popliteal artery (5 cases), and superficial femoral artery (4 cases). Venous thrombosis (21 cases, 30.4%) was also commonly seen in these patients, including superficial femoral vein (9 cases), popliteal vein (9 cases), common femoral vein (7 cases), posterior tibial vein (6 cases), and external iliac vein (4 cases). Arterial thrombosis (9 cases, 13.0%, majorly involve abdominal aorta and superficial femoral artery) and venous stenosis or occlusion (8 cases, 11.6%, majorly involve superior vena cava and inferior vena cava) were also documented in these patients.

### 3.3. BD Manifestations

For the other BD manifestations, oral ulceration was presented in all patients, followed by skin lesions (76.8%), genital ulceration (69.6%), and pathergy reaction (44.9%). Ocular (14.5%), cardiac (13.0%) (intracardiac thrombosis, valvular disease, and myocardial involvement in 2 cases, respectively, and pericarditis in 1 case), gastrointestinal (11.6%), and neurological (8.7%) involvements were less frequently presented. No significant difference in these manifestations between BD patients with pulmonary aneurysms and those with aortic/peripheral artery aneurysmal lesions was observed. The comparison of the clinical characteristics of the BD patients with and without aneurysmal lesions is listed in Table 2.

### 3.4. Laboratory Examinations

Inflammatory markers were significantly elevated in 57 patients (82.6%), suggesting the development of aneurysmal lesions. The mean erythrocyte sedimentation rate (ESR) was 36.1 ± 23.8 mm/hr, and the median C-reactive protein (CRP) level was 32.2 (0.2-168.9) mg/dl. Mean ESR and median CRP level between BD patients with pulmonary aneurysms and those with aortic/peripheral artery aneurysmal lesions were comparable.

### 3.5. Risk Factors for Developing Aneurysmal Lesions in BD Patients

According to univariate analysis, factors significantly associated with the complication of aneurysmal lesions in BD patients include pathergy reaction (*P* < 0.001), arterial stenosis or occlusion (*P* < 0.001), arterial thrombosis (*P* = 0.002), venous involvement (*P* = 0.001), venous thrombosis (*P* = 0.028), elevated ESR levels (*P* < 0.001), and elevated CRP levels (*P* < 0.001). Ocular involvement (*P* = 0.032) and gastrointestinal involvement (*P* = 0.022) showed a negative correlation with aneurysmal formation.

Furthermore, the multivariate logistic regression analysis revealed that BD patients with pathergy reaction (OR = 3.78, 95% CI 1.70-8.41), arterial stenosis or occlusion (OR = 44.12, 95% CI 11.56-168.35), and arterial thrombosis (OR = 9.27, 95% CI 2.33-36.93) had a significantly increased risk of developing aneurysmal lesions (Table 3).

### 3.6. Treatment

At the onset of aneurysmal lesions, 59 patients (85.5%) were glucocorticoids (GCs) or immunosuppressant treatment-naïve; the other 10 (14.5%) patients have received the low-to-medium dose of GCs (≤ 30 mg/d prednisone or equivalent).

After the diagnosis of aneurysmal lesions, 66 patients (95.6%) were treated with GCs, including 7 patients (10.1%) with methylprednisolone pulse therapy, 48 patients (69.6%) with the large dose of GCs, and 11 patients (15.9%) with the low-to-medium dose of GCs, and the dose was gradually tapered over several weeks. Cyclophosphamide, the first-line choice of immunosuppressant, was used in 60 patients (87.0%); others received immunosuppressants including azathioprine, cyclosporine, methotrexate, leflunomide, and mycophenolate mofetil. Furthermore, 19 patients (27.5%) received a combination of 2 or more immunosuppressants. Five severe and/or refractory cases (7.2%) received biological agents, including adalimumab (ADA) in 2 cases and tocilizumab (TCZ), infliximab (IFX), and TCZ followed by IFX in 1 case, respectively, and all proved effective with no newly-onset aneurysmal lesions.

Forty-one patients (59.4%) underwent interventional procedures or open operation. For 10 patients with PAA, 5 patients took interventional embolization and 1 patient received pulmonary lobectomy. For other 59 patients with aortic and/or peripheral aneurysmal lesions, 16 patients received endovascular graft deployment, 2 patients took interventional embolization, 9 patients received surgical aneurysmectomy with or without bypass surgery, and combination therapies were applied in other 8 patients.

### 3.7. Outcome

After a mean follow-up of 5.5 ± 4.0 years, 10 patients (the mortality rate was 14.5%) died and 9 patients (13.0%) lost to follow-up. Among the 10 death cases, 5 (50%) patients relapsed, including worsening of the preexisting aneurysmal lesions (*n* = 3) and the occurrence of new aneurysmal lesions (*n* = 2). The overall respective estimated 1- and 5-year survival rates were 95.0% and 87.2%. Rupture of the pulmonary artery and/or coronary artery involvement was the main cause of death which occurred in 6 patients (Table 4).

**Figure 2:**
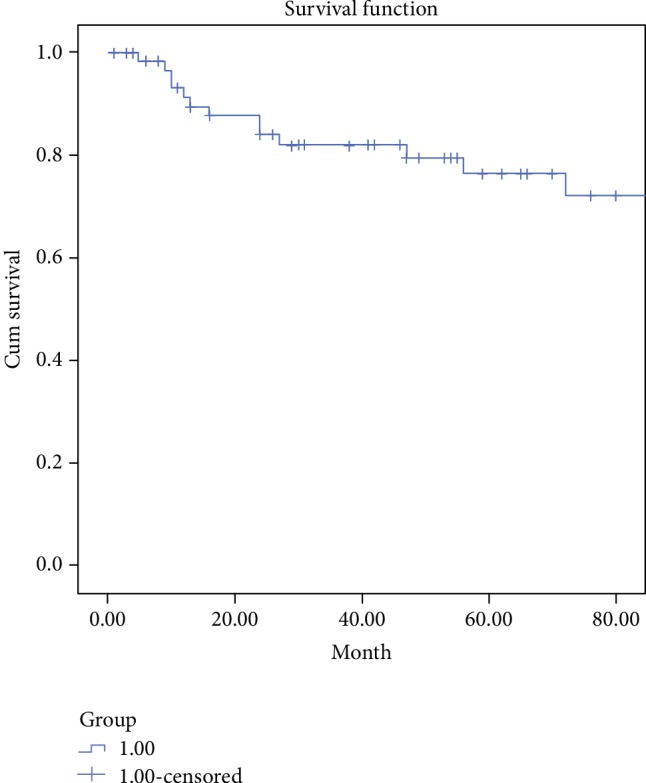
Kaplan-Meier survival analysis showing cumulative relapse-free rates of the disease in a cohort of 69 BD patients with aneurysmal lesions.

For the rest of the patients, the other 10 patients relapsed during follow-up, including worsening of the preexisting aneurysmal lesions (*n* = 6), the occurrence of new aneurysmal lesions (*n* = 3), and arterial stenosis (*n* = 1). The respective estimated cumulative 1- and 5-year relapse-free rates were 91.3% and 76.3% (Figure 2). For all the patients who underwent endovascular graft deployment, 2 patients developed in-stent restenosis, 1 patient had stent thrombosis, and 2 patients had the recurrence of the abdominal aorta aneurysm nearby the stent within a year after the first interventional therapy. One patient had the left thigh abscess 7 years after the interventional therapy and had to do surgery to remove the stent at last.

Three patients underwent a second interventional therapy during follow-up. Among them, one patient developed a new pseudoaneurysm in the abdominal aorta after 6 years of follow-up, and an endovascular graft deployment was performed. As for the other 2 patients mentioned above who had the recurrence of the aneurysm in the abdominal aorta aneurysm nearby the stent, one patient received a second endovascular repair by graft deployment and the other patient underwent an interventional embolization.

Forty patients (58.0%) achieved clinical remission after treatment. The clinical remission was defined as the absence of newly-onset arterial lesions or the progression of the preexisting vascular lesion due to BD, with normalized ESR and CRP.

## 4. Discussion

We report a retrospective study on 69 Chinese BD patients complicated with aneurysmal lesions and provide a thorough analysis of the clinical characteristics, treatment, and outcome of these patients. The results suggested the complexity of vascular involvement and poor prognosis in BD patients with aneurysmal lesions. We also identified factors predicting the development of aneurysmal lesions in BD patients. To the best of our knowledge, this study is the largest and first cohort study reporting the risk factors of aneurysmal lesions in BD patients.

The vascular lesion is one of the most important manifestations of BD patients. In the previous studies [2, 8], arterial involvement was reported in 7.0-12.3% of BD patients. As reported in a previous study from South Korea [9], the frequency of aneurysmal lesions in BD patients is very rare. 0.94% of BD patients had true aneurysm and pseudoaneurysm in the major artery system. Our study shows that the development of aneurysmal lesions tends to occur in the early phase of BD, with male predominance, and most of the patients have not received glucocorticoids or immunosuppressants before the onset of aneurysmal lesions. Previous studies reported that positive pathergy reaction showed a significant association with vascular lesions [10, 11]. Similarly, multivariate analysis in this study shows that pathergy reaction is the independent risk factor of aneurysmal lesion formation in BD patients. We find that arterial stenosis or occlusion and arterial thrombosis are predictive factors of aneurysmal lesions in BD patients, which indicates that physical and imaging examinations should be performed to exclude aneurysmal lesions in BD patients if they have other types of arterial involvements.

In patients with aortic and/or peripheral artery aneurysms, the sequentially preferential location of the aneurysmal lesion is the abdominal aorta, coronary artery, superficial femoral artery, subclavian artery, and popliteal artery, which accounts for 56.7% (55/97) of the entire aortic and/or peripheral artery aneurysms. A predominance of pseudoaneurysms for aortic and/or peripheral artery involvement was shown in our study. Previous studies [9, 12] showed that 30-40% of BD patients with aneurysmal lesions had 2 or more aneurysmal lesions. Consistently, nearly half of the patients in our cohort had 2 or more aneurysmal lesions. Most of the BD patients with coronary artery lesions present acute coronary syndrome at a very early age. Some of these patients also had coronary artery stenosis/occlusion, which also might be an underlying cause of acute coronary syndrome. All patients with pulmonary artery involvement had hemoptysis. In a French study [13], hemoptysis was the inguinal symptom in 14 out of 17 patients with pulmonary artery aneurysm. As acute coronary syndrome and hemoptysis can both be severe and life-threatening, early diagnosis and appropriate treatment are required.

Glucocorticoids and cyclophosphamide are the basic treatment for BD patients with aneurysmal lesions [8, 14, 15] according to the 2018 update of the European League Against Rheumatism (EULAR) recommendations [16]. Several studies, most of which are anecdotal reports and small case series, have reported that anti-TNF-*α* therapy is effective in severe and/or refractory BD patients with aneurysmal lesions [17–20] and thus was recommended by EULAR [16]. In our study, 4 refractory cases were treated with monoclonal anti-TNF-agents, and all patients showed responsiveness during follow-up. However, in real clinical practice, the uses of TNF-*α* inhibitors are limited by the inadequate response, secondary loss of efficacy, intolerance, and contraindications. Thus, it is necessary to find alternative therapeutics for severe and/or refractory BD. In our study, TCZ was applied to one patient with recurrent arterial pseudoaneurysms and concurrent suspicious tuberculosis pericarditis, which achieved both clinical and serological improvements. Further prospective controlled studies are warranted to investigate the therapeutic potential of TCZ in treating vascular BD.

The common surgical and interventional procedures involved in this group of patients include endovascular graft deployment, interventional embolization, and aneurysmectomy with or without bypass surgery. In BD patients with PAA, interventional embolization was commonly used. In BD patients with aortic and/or peripheral aneurysmal lesions, endovascular graft deployment was the preferred interventional procedure. However, BD patients with aneurysmal lesions remain challenging for surgeons due to the vascular inflammation that increases the difficulties of the procedure and the risks of postoperative complications [21]. In our study, most patients responded well after interventional or surgical procedures, but there were still a few complications after procedures, including infection, recurrence of aneurysmal lesions, in-stent restenosis, and stent thrombosis. Moreover, 3 patients underwent a second interventional therapy. Kalko et al. [22] reported that BD patients who achieved remission before surgery could have a decreased incidence of postoperative complications. As a consequence, appropriate evaluation before the first intervention, ideal postoperative management with immunosuppressive therapy may reduce the incidence of postoperative complications and prevent the repeat procedures for BD patients with aneurysmal lesions.

Saadoun et al. [8] had reported 101 BD patients with arterial lesions in which 47.3% of arterial involvement were aneurysms, and 14 patients died during follow-up. Two other reports [22, 23] showed that the mortality rate is relatively low in BD patients with nonpulmonary aneurysms, but they still had a high rate of relapse. Our study also indicates the poor prognosis of this condition. The causes of death in our study are mainly due to aneurysmal lesions or the accompanying vascular lesions, especially in patients with pulmonary or coronary artery involved.

There are several limitations in our study. It is a retrospective study from a single center. Also, some patients lost to follow-up, which affected the outcome analysis. It is important to conduct prospective cohorts with controls and elaborate follow-up to establish more findings in the future.

In conclusion, aneurysmal lesions tend to happen in the early phase of BD and can lead to severe and life-threatening condition. Pathergy reaction, arterial stenosis or occlusion, and arterial thrombosis are the risk factors of aneurysmal lesions in BD patients. Achieving BD remission and performing surgical or interventional procedures are both important in the treatment of these patients.

## Figures and Tables

**Table 1:** The site, number, and type of aortic and/or peripheral artery aneurysmal lesions in BD patients.

Site	True aneurysm	Pseudoaneurysm	Unclassified	Total
Abdominal aorta	8	19		27
Common femoral artery		4		4
Deep femoral artery		3		3
Superficial femoral artery	1	6	1	8
Ascending thoracic aorta		4		4
Descending thoracic aorta	1	2	1	4
Aortic arch		2	1	3
Coronary artery	8	2		10
Common iliac artery	2	2		4
External iliac artery		4		4
Internal iliac artery		1		1
Subclavian artery	1	4		5
Popliteal artery		5		5
Carotid artery	3		1	4
Lower leg artery	1	3		4
Superior mesenteric artery	1	1		2
Innominate artery		2		2
Ileocolic artery		1		1
Celiac trunk		1		1
Ulnar artery		1		1

**Table 2:** Demographic and clinical characteristics of BD patients with and without aneurysmal lesions.

Clinical features	Aneurysmal group (*n* = 69)	Nonaneurysmal group (*n* = 207)	*P* value
Age at registration (years)	36.0 ± 11.6	35.5 ± 12.2	0.788
Gender (male (%))	56 (81.2%)	168 (81.2%)	1.000
Oral ulceration	69 (100%)	207 (100%)	NA
Genital ulceration	48 (69.6%)	147 (71%)	0.819
Pseudofolliculitis	21 (30.4%)	78 (37.7%)	0.277
Erythema nodosum	31 (44.9%)	85 (41.1%)	0.573
Pathergy reaction	31 (44.9%)	38 (18.4%)	<0.001
Ocular involvement	10 (14.5%)	57 (27.5%)	0.032
Gastrointestinal involvement	8 (11.6%)	52 (25.1%)	0.018
Arterial stenosis or occlusion^#^	26 (37.7%)	3 (1.4%)	<0.001
Arterial thrombosis	9 (13.0%)	5 (2.4%)	0.002
Venous involvement	26 (37.7%)	37 (17.9%)	0.001
Venous thrombosis^∗^	21 (30.4%)	37 (17.9%)	0.027
CNS involvement	4 (5.8%)	11 (5.3%)	1.000
BDCAF2006 score	4.75 ± 2.03	5.16 ± 2.03	0.151
ESR	36.1 ± 23.8	21.2 ± 21.7	<0.001
CRP, median (range)	32.2 (0.2-168.9)	6.3 (0.1-239.6)	<0.001

NA: not applied. ^#^The sequentially preferential location of arterial stenosis or occlusion included subclavian artery, external iliac artery, celiac truck and coronary artery. ^∗^The sequentially preferential location of venous thrombosis included superficial femoral vein, popliteal vein, common femoral vein, and posterior tibial vein.

**Table 3:** Univariate and multivariate analysis of clinical and laboratory findings for 69 BD patients with aneurysmal lesions and 207 controls.

	Univariate (unadjusted)		Multivariate (adjusted)	
	OR (95% CI)	*P* value	OR (95% CI)	*P* value
Pathergy reaction	3.63 (2.01-6.55)	<0.001	3.78 (1.70-8.41)	0.001
Ocular involvement	0.45 (0.21-0.93)	0.032	NS	NS
Gastrointestinal involvement	0.39 (0.18-0.87)	0.022	NS	NS
Arterial stenosis or occlusion	41.12 (11.90-142.01)	<0.001	44.12 (11.56-168.35)	<0.001
Arterial thrombosis	6.06 (1.96-18.77)	0.002	9.27 (2.33-36.93)	0.002
Venous involvement	2.78 (1.52-5.08)	0.001	1.78 (0.76-4.15)	0.182
Venous thrombosis	2.01 (1.08-3.75)	0.028	NS	NS
ESR	1.03 (1.01-1.04)	<0.001	1.02 (1.00-1.04)	0.069
CRP, median (range)	1.02 (1.01-1.03)	<0.001	1.01 (0.99-1.02)	0.25

OR: odds ratio; NS: not selected.

**Table 4:** Outcome of 69 BD patients with aneurysmal lesions.

Outcome	*N* (total = 69)
Clinical improvement	40 (58.0%)
Relapse	15 (21.7%)
Worsening of the original aneurysmal lesions	9
Newly-onset aneurysmal lesions	5
Newly-onset arterial stenosis	1
Death	10 (14.5%)
Hemoptysis	3
Acute myocardial infarction	2
Cardiac arrest	1
Rupture of the dissecting aneurysm in the abdominal aorta	1
Unknown causes	3
Lost to follow-up	9 (13.0%)

## Data Availability

The clinical data used to support the findings of this study are included in the article.
